# Preanalytics of urine sediment examination: effect of relative centrifugal force, tube type, volume of sample and supernatant removal

**DOI:** 10.11613/BM.2018.010707

**Published:** 2018-01-10

**Authors:** Amalija Bunjevac, Nora Nikolac Gabaj, Marijana Miler, Anita Horvat

**Affiliations:** 1 Department of laboratory diagnostics, Children’s hospital Zagreb, Zagreb, Croatia; 2 Department of Clinical Chemistry, Sestre milosrdnice University Hospital Center, Zagreb, Croatia; * Corresponding author: amalijabunjevac@gmail.com

**Keywords:** preanalytical phase, laboratory error, urinalysis, urine sediment

## Abstract

**Introduction:**

Laboratories often modify procedures recommended by the European Urinalysis Guidelines for urine sediment analysis. The aim of this study was to compare the recommended protocol with our routine laboratory procedure and to evaluate the possible impact of modifications in the relative centrifugal force, type of tube, method of supernatant aspiration and urine volume on patient’s results.

**Material and methods:**

Firstly, relative centrifugal force was investigated using 20 pairs of samples examined after centrifugation at 400xg and 1358xg. In phase two, 110 samples were examined, paired as: round bottom *vs* conical tube (N = 46), decanting *vs* suction of supernatant (N = 100) and 10 mL *vs* 5 mL of urine sample (N = 101).

**Results:**

The number of erythrocytes, leukocytes and squamous epithelial cells was significantly lower after centrifugation at 400xg (P = 0.001, 0.002 and 0.004, respectively). The number of leukocytes was significantly lower in conical tubes (P = 0.010), after the suction of supernatant (P = 0.045) and in 5 mL urine (P < 0.001). The number of squamous epithelial cells was significantly lower after the suction of supernatant (P < 0.001) and in 5 mL urine (P < 0.001). The number of erythrocytes (P < 0.001), total non-hyaline casts (P < 0.001) and the frequency of granular casts (P = 0.039) was significantly lower in 5 mL urine.

**Conclusion:**

Lower results of leukocytes, erythrocytes, squamous cells and non-hyaline casts were recorded in recommended procedures (centrifugation at 400xg, suction of supernatant, conical tube, 5 mL of sample) than in routine procedure (centrifugation at 1358xg, decanting of supernatant, round bottom tube, 10 mL) used in our laboratory.

## Introduction

Urinalysis is an integral part of routine laboratory work. Qualitative urinalysis includes visual inspection of urine, chemical analysis and microscopic analysis of urinary sediment (*1*). Elements of urine sediment are divided in two groups, organized and unorganized elements. Organized urine sediment consists of biological elements such as leukocytes, erythrocytes, epithelial cells, casts, bacteria, fungi, parasites and sperm. Unorganized urine sediment contains crystals of various salts, for instance oxalate, phosphate, urate, and amorphous salts. Components of urinary sediment, especially casts, have a great clinical significance in diagnosis and management of renal patients. Great expertise, years of practice and vast experience are required for accurate identification and classification of urine sediment elements (*2*).

Manual urine sediment analysis was a gold standard in laboratory work for decades (*3*, *4*). In most laboratories, a bright-field microscopy of unstained centrifuged native urine is still a part of routine work. However, detailed protocols, especially in the preanalytical phase, slightly vary between laboratories (*3*). There is no reference method for urine sediment microscopy (*1*, *4*).

The preanalytical phase is the most vulnerable part of laboratory process accounting for up to 75% of all laboratory errors (*4*-*8*). The preanalytical phase is comprised of several sub-phases: need for the test, patient preparation, sample collection, sample transport and preparation of sample for testing (*4*, *8*). Even though the laboratory is not directly involved in all those steps, laboratory staff is responsible for their correct execution (*9*). For example, providing detailed user-friendly instructions for patients regarding preparation and correct method of sample collection or educating non-laboratory staff involved in sample transport on exact conditions in which samples should be stored. Efficient patient preparation is crucial for gaining an adequate urine sample (*1*, *4*). The preparation of samples for testing, principally centrifugation efficiency and residual volume of the sediment, has been shown to be a large source of errors in the preanalytical phase (*4*).

In order to standardize urinalysis, the European Confederation of Laboratory Medicine has published the European Urinalysis Guidelines, which provide specific instructions for urinary sediment analysis (*1*).

Based on the availability of the equipment, consumables, materials and reagents, laboratories often include some modifications to the recommended protocol, based on the local specificities. However, prior to introduction into routine practice, the possible impact on the results of laboratory tests has to be investigated. The protocol used for urinalysis in the Department of Clinical Chemistry, Sestre Milosrdnice University Hospital Center, Zagreb, Croatia, includes some modifications with regards to the recommendations given in the European Urinalysis Guidelines. These modifications include centrifugation speed, type of tube, minimal volume required for testing and method of aspiration of supernatant. Therefore, the aim of our study was to investigate whether there is a difference in the results of urine sediment microscopy of routine protocol used in our laboratory comparing to the recommended protocol by the European Urinalysis Guidelines.

## Materials and methods

### European Urinalysis Guidelines

Requirements for urine sediment examination and modifications used in the Department of Clinical Chemistry, Sestre Milosrdnice University Hospital Center, Zagreb, Croatia (routine procedure) are presented in Table 1.

**Table 1:** Requirements for urine sediment examination according to the European Urinalysis guidelines and modifications of routine laboratory procedure

	**European Urinalysis Guidelines**	**Routine** **procedure**
**Sample volume, mL**	5 - 12	5 - 10
**Tube**	Conical bottom tube	Round bottom tube
**Centrifugation**	400xg for 5 min, preferably at 4 °C if delayed	1358xg for 10 min
**Supernatant removal**	Suction with an adjusted vacuum tool	Decanting
**Staining and microscopy**	Phase-contrast microscopy, or staining with bright-field microscopy; polarized optics when needed; low (x100) and high-power magnification (x400)	Bright-field microscopy of unstained preparations; low (x100) and high-power magnification (x400)

### Study design

This study was conducted in the Department of Clinical Chemistry, Sestre Milosrdnice University Hospital Center, Zagreb, Croatia. We investigated leftover routine urine samples collected in the Department of Nephrology of the Internal Disease Clinic. Samples were delivered to the laboratory in urine collection cups (Urine Beaker with integrated Transfer Device 100 mL, Greiner Bio-One, Kremsmuenster, Austria).

This study was performed in two phases. In the first phase, which was done in 2015, we investigated the relative centrifugal force (RCF) modification. In the second phase, carried out from March to April 2016, three other modifications were investigated.

For the investigation of RCF modification, 20 urine samples were compared. Each sample was divided in two round bottom urine sample tubes (Vacuette® tube 10 mL Z Urine No Additive, 16x100 yellow cap-yellow ring, Round Base, non-ridged; Greiner Bio-One, Kremsmuenster, Austria) and then centrifuged at 400xg according to the Guidelines and at 1358xg according to the routine procedure. The supernatants were decanted and the sediments were resuspended and examined under microscope (Opton Standard Zeiss, Oberkochen, Germany). To minimize inter-individual variation all samples were examined using the same microscope by one skilled laboratory technician (bachelor of medical laboratory diagnostics). This phase was done initially to determine which RCF will be used in the phase 2 of the study.

**Figure 1:**
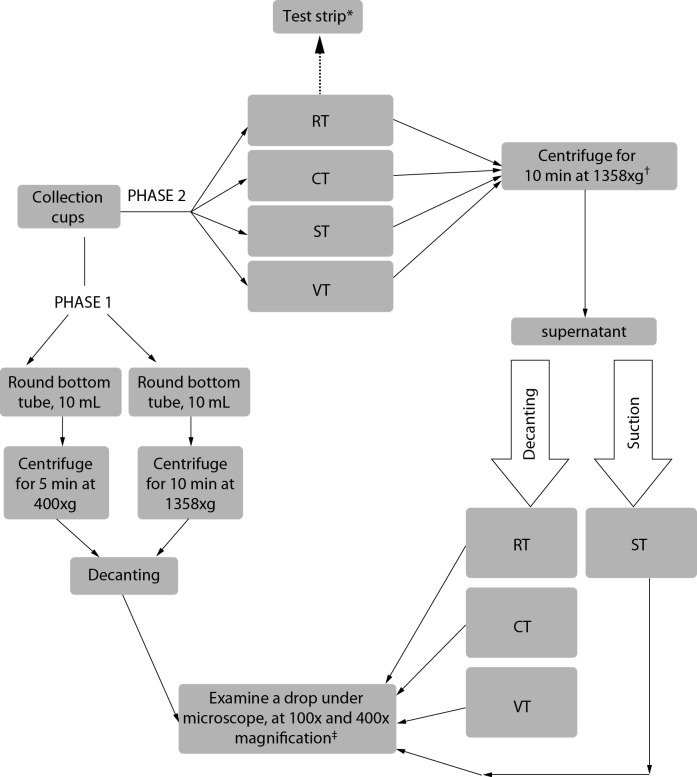
Study protocol of investigation modifications in urine sediment examination. *Cobas u411 (Roche Diagnostics, Basel, Switzerland). ^†^Rotofix 32A (Hettich, Tuttlingen, Germany). ^‡^Opton Standard (Zeiss, Oberkochen, Germany). RT- round bottom tube, decanting, 10 mL. CT- conical bottom tube, decanting, 10 mL. ST- round bottom tube, suction, 10 mL. VT- round bottom tube, decanting, 5 mL.

For other modifications, a total of 110 samples were collected. Because of the sample quantity: 47 samples were used for tube comparison, 100 were used for comparing methods of supernatant removal and 101 were used for sample volume comparison. From every sample one round bottom tube (RT - routine tube) was analyzed using the laboratory routine procedure. Depending on the residual sample volume, the urine sample was divided into 3 additional tubes, one conical bottom (CT - conical tube; Vacuette® tube 9.5 mL Z Urine No Additive, 16x100 yellow cap-yellow ring, Conical Base, non-ridged; Greiner Bio-One, Kremsmuenster, Austria) and two round bottom (ST-suction tube, VT-volume tube). Urine samples with inadequate volume for at least one additional full tube were excluded. All tubes were then centrifuged for 10 min at 1358xg using the Rotofix 32A (Hettich, Tuttlingen, Germany) (based on the results of the first phase of the study). Supernatant was decanted or removed by disposable plastic pipettes (Samco™ Transfer Pipettes, Thermo Fisher Scientific, Waltham, USA). Urine sediment was then resuspended in 500 μL of supernatant and a 10 μL drop was examined under the microscope (Opton Standard Zeiss, Oberkochen, Germany). To minimize inter-individual variation, all samples were examined using the same microscope by one skilled laboratory technician (bachelor of medical laboratory diagnostics) (Figure 1).

### Statistical analysis

Normality of distribution for quantitative data was tested with D’Agostino-Pearson test. All parameters were non-normally distributed; therefore they were described by median and interquartile range (IQR). Wilcoxon signed rank test, a non-parametric paired test was used to test the difference in number of elements between test tubes for quantitative data (erythrocytes, leukocytes, squamous epithelia cells; based on modification hyaline or non-hyaline casts *per* objective field).

Sample size was limited by the number of samples with appropriate volume received in the laboratory during the period of the study. Minimal sample size for each comparison was set at 20 samples. Due to the small number of elements found, some parameters couldn’t be tested as quantitative data so they were categorized in two categories, no elements found *vs* at least one element found. These data (leukocytes in clusters, small squamous cells, granular casts and based on modification hyaline or non-hyaline casts *per* objective field) were presented as N/total and tested by McNemar’s exact tests (*10*).

For bacteria, mucus and fungi, which were described with ordinal scale (0, 1+, 2+, 3+) an inter-rater agreement was determined using Cohen’s kappa coefficient and corresponding 95% confidence interval (95% CI). P < 0.05 was considered statistically significant. Kappa coefficient was considered acceptable if lower limit of 95% confidence interval was higher than 0.6 (*11*).

Statistical analysis was performed with MedCalc, Version 11.5.1 (MedCalc softver, Ostend, Belgium) for D’Agostino-Pearson test, Wilcoxon signed rank test and Cohen’s kappa coefficient while Statistical program R, package version 3.4.2 (2017, Vienna, Austria) was used for analyzing the data with McNemar’s exact test (*12*).

## Results

### Phase 1 - RCF modification

Results of evaluation of RCF modification are presented in Table 2. For all quantitative parameters (number of leukocytes, erythrocytes and squamous epithelia cells *per* objective field), significantly lower values were observed when urine sediment was centrifuged at 400xg (P = 0.001, 0.002 and 0.004, respectively).

**Table 2:** Elements of urine sediment according to different relative centrifugal force

**Element**	**Relative centrifugal force 1358xg** **N = 20**	**Relative centrifugal force 400xg** **N = 20**	P*
Erc, HF	2 (1 - 8)	1 (0 - 3)	**0.002**
Lkc, HF	9 (3 - 21)	3 (1 - 9)	**0.001**
Squamous epithelial cells, HF	6 (3 - 9)	2 (1 - 8)	**0.004**
Non-hyaline casts, LF	2/20	1/20	1.000
Lkc in clusters, HF	9/20	7/20	0.500
Small squamous cells, HF	10/20	10/20	1.000
Hyaline casts, LF	2/20	0/20	0.500
Granular casts, LF	2/20	0/20	0.500
Bacteria, HF			
0	7/20	9/20	**0.64** **(0.42 - 0.87)**
1+	2/20	5/20	
2+	8/20	3/20	
3+	3/20	3/20	
Mucus, HF			
0	3/20	13/20	**0.20** **(-0.01 - 0.42)**
1+	6/20	2/20	
2+	11/20	5/20	
Fungi, HF			
0	20/20	20/20	/
Quantitative data are presented as median and interquartile range; qualitative data are presented as N/total. *P-values for quantitative data are calculated using Wilcoxon’s test, for qualitative data with McNemar’s exact test; kappa coefficient with 95% confidence interval is calculated for bacteria, mucus and fungi. Statistically significant differences are highlighted in bold. Erc – erythrocytes. Lkc – leukocytes. HF – *per* high power objective field. LP – *per* low power objective field.			

There was no statistically significant difference in frequency of found casts, leukocytes in clusters and small squamous cells between two RCFs.

Cohen’s kappa coefficient (κ) was calculated for bacteria and mucus, since fungi were not observed in this set of samples. Agreement was not acceptable for both parameters: bacteria (κ (95% CI) = 0.64 (0.42 - 0.87)) and mucus (κ (95% CI) = 0.20 (-0.01 - 0.42)). For both parameters, lower level of positive results was recorded when samples were centrifuged at 400xg.

### Phase 2 - tube type modification and aspiration of supernatant modification

Results for type of tube comparison are presented in Table 3. For the number of leukocytes, significantly lower values were observed in conical tube than in round bottom tube (P = 0.010), while there was no statistically significant difference in the number of erythrocytes, squamous epithelial cells and total non-hyaline casts. Frequency of leukocytes in clusters, small squamous cells, hyaline casts and granular casts also did not differ between two tube types. Agreement for bacteria, mucus and fungi was acceptable between round bottom and conical tube (κ (95% CI) = 0.93 (0.85 - 1.00); κ (95% CI) = 0.76 (0.64 - 0.91); κ (95% CI) = 1.00 (1.00 - 1.00); respectively).

**Table 3:** Elements of urine sediment according to tube type

**Element**	**RT tube, N = 47**	**CT tube, N = 47**	P*
Erc, HF	4 (2 - 11)	3 (1 - 8)	0.054
Lkc, HF	5 (2 - 15)	3 (2 - 18)	**0.010**
Squamous epithelial cells, HF	3 (2 - 10)	2 (1 - 8)	0.343
Non-hyaline cast, LF	0 (0 - 2)	0 (0 - 1)	0.054
Lkc in clusters, HF	13/47	16/47	0.508
Small squamous cells, HF	8/47	8/47	1.000
Hyaline casts, LF	6/47	8/47	0.688
Granular casts, LF	18/47	12/47	0.063
Bacteria, HF			
0	12/47	12/47	0.93 (0.85 - 1.00)
1+	13/47	14/47	
2+	10/47	8/47	
3+	12/47	13/47	
Mucus, HF			
0	33/47	17/47	0.76 (0.64 - 0.91)
1+	22/47	15/47	
2+	12/47	15/47	
Fungi, HF			
0	46/47	46/47	1.00 (1.00 - 1.00)
1+	1/47	1/47	
Quantitative data are presented as median and interquartile range; qualitative data are presented as N/total. *P-values for quantitative data are calculated using Wilcoxon’s test, for qualitative data with McNemar’s exact test; kappa coefficient with 95% confidence interval is calculated for bacteria, mucus and fungi. Statistically significant differences are highlighted in bold. RT - round bottom tube, suction, 10 mL. CT - conical bottom tube, decanting, 10 mL. Erc – erythrocytes. Lkc – leukocytes. HF – *per* high power objective field. LP – *per* low power objective field.			

Results of the comparison of supernatant aspiration method are presented in Table 4. For the number of leukocytes and squamous epithelial cells, significantly lower values were observed when supernatant was removed by suction (P = 0.045 and P < 0.001, respectively). There was no statistically significant difference in the number of erythrocytes, total non-hyaline casts, and leukocytes in clusters, small squamous cells, hyaline casts and granular casts between the two methods of supernatant aspiration. Degree of agreement was strong for bacteria and fungi, but it was not acceptable for mucus (κ (95% CI) = 0.681 (0.567 – 0.795). Lower frequency of positive results was observed when supernatant was removed by suction.

**Table 4:** Elements of urine sediment according to different method of supernatant aspiration

**Element**	**RT, N = 101**	**ST, N = 101**	P*
Erc, HF	4 (2 - 11)	4 (1 - 9)	0.150
Lkc, HF	5 (2 - 15)	4 (2 - 8)	**0.045**
Squamous epithelial cells, HF	3 (2 - 10)	3 (1 - 10)	**< 0.001**
Non-hyaline cast, LF	0 (0 - 2)	0 (0 - 2)	0.100
Lkc in clusters, HF	27/101	26/101	1.000
Small squamous cells, HF	17/101	16/101	1.000
Hyaline casts, LF	19/101	17/101	0.791
Granular casts, LF	41/101	39/101	0.754
Bacteria, HF			
0	62/101	38/101	0.88 (0.82 - 0.94)
1+	34/101	21/101	
2+	22/101	26/101	
3+	5/101	38/101	
Mucus, HF			
0	31/101	46/101	**0.68 (0.57 - 0.80)**
1+	52/101	37/101	
2+	18/101	18/101	
Fungi, HF			
0	95/101	95/101	1.00 (1.00 - 1.00)
1+	6/101	6/101	
Quantitative data are presented as median and interquartile range; qualitative data are presented as N/total. *P-values for quantitative data are calculated using Wilcoxon’s test, for qualitative data with McNemar’s exact test; kappa coefficient with 95% confidence interval is calculated for bacteria, mucus and fungi. Statistically significant differences are highlighted in bold. RT - round bottom tube, decanting, 10 mL. ST - round bottom tube, suction, 10 mL. Erc – erythrocytes. Lkc – leukocytes. HF – *per* high power objective field. LP – *per* low power objective field.			

Results of comparison of sample volume are presented in Table 5. Significantly lower values are observed when 5 mL of urine was analyzed for the number of leukocytes, erythrocytes, squamous epithelial cells and total non-hyaline casts (P < 0.001 for all). Furthermore, granular casts were also observed with lower frequency in 5 mL samples than in 10 mL (P = 0.039), while there was no statistically significant difference in frequency of leukocytes in clusters, small squamous cells and hyaline casts between 5 mL samples and 10 mL samples. Degree of agreement was strong for bacteria and fungi, but it was not acceptable for mucus (κ (95% CI) = 0.656 (0.544 – 0.769)). Lower frequency of positive results was observed in the 5 mL tube.

**Table 5:** Elements of urine sediment according to different minimal volume required for testing

**Element**	**RT, N = 100**	**VT, N = 100**	P*
Erc, HF	4 (2 - 11)	2 (1 - 7)	**< 0.001**
Lkc, HF	5 (2 - 15)	3 (1 - 8)	**< 0.001**
Squamous epithelial cells, HF	3 (2 - 10)	2 (1 - 6)	**< 0.001**
Non-hyaline cast, LF	0 (0 - 2)	0 (0 - 1)	**< 0.001**
Lkc in clusters, HF	28/100	24/100	0.481
Small squamous cells, HF	19/100	13/100	0.180
Hyaline casts, LF	17/100	11/100	0.146
Granular casts, LF	48/100	34/100	**0.039**
Bacteria, HF			
0	29/100	34/100	0.93 (0.88 - 0.97)
1+	29/100	24/100	
2+	21/100	23/100	
3+	21/100	19/100	
Mucus, HF			
0	34/100	52/100	**0.66 (0.54 - 0.77)**
1+	45/100	31/100	
2+	21/100	17/100	
Fungi, HF			
0	94/100	95/100	0.90 (0.72 - 1.00)
1+	6/100	5/100	
Quantitative data are presented as median and interquartile range; qualitative data are presented as N/total. *P-values for quantitative data are calculated using Wilcoxon’s test, for qualitative data with McNemar’s exact test; kappa coefficient with 95% confidence interval is calculated for bacteria, mucus and fungi. Statistically significant differences are highlighted in bold. RT - round bottom tube, decanting, 10 mL. VT - round bottom tube, decanting, 5 mL. Erc – erythrocytes. Lkc – leukocytes. HF – *per* high power objective field. LP – *per* low power objective field.			

## Discussion

This study shows significant differences in urine sediment results for leukocytes, erythrocytes and squamous cells between the procedure recommended by European Urinalysis Guidelines and the routine protocol performed in our laboratory. Modifications in relative centrifugation force, tube type, sample volume and supernatant removal affect results of urine sediment examination.

Investigation of relative centrifugal force confirms that the number of erythrocytes, leukocytes and squamous epithelial cells *per* objective field is significantly lower after the centrifugation at 400xg than after the centrifugation at 1358xg. Pyuria and haematuria are a major sign of renal and urinary tract disease, therefore a false-negative number of erythrocytes and leukocytes can prolong diagnosis of urinary tract disease and accordingly cause harm for patients (*1*, *13*, *14*). Because our samples came from hospitalized patients with renal and/or urinary tract disease, higher number of erythrocytes and leukocytes were expected. Our consultations with the clinical staff at the nephrology department confirmed that higher numbers of those elements are correspondent with clinical presentation of the disease, while the lower numbers do not match the patient condition. Small number of leukocytes and erythrocytes *per* objective field could be found in urine sediment of healthy patients due to exercise or pregnancy (*15*, *16*). Errors in preanalytical phase during urine collection are fairly common and can lead to the contamination of urine sample with erythrocytes or leukocytes (*e.g.* incorrect mid-stream collection without proper outer genital tract hygiene) (*4*, *17*). In accordance with this, falsely low number of erythrocytes and leukocytes observed after the centrifugation at 400xg can then be misinterpreted as preanalytical error or physiological presence.

Results of RCF investigation confirmed our empirical observation that the relative centrifugal force of 400xg is not enough for adequate urine sediment preparation. According to these results it was determined that all samples in phase 2 of this study should be centrifuged at 1358xg. There were no disadvantages of centrifugation at 1358xg.

When urine sediments were analyzed in routinely used round bottom tube and recommended conical tube, number of leukocytes was significantly lower in the conical tube. We suspect that the cause of this unexpected result lies in the material of the sample tube. In the past, sample tubes were made of glass which had almost perfect adhesive characteristics, but nowadays all tubes are plastic. It was previously published that different types of plastic blood collection tubes could interfere with blood test results (*18*). However, adhesive performance of plastic urine tubes is still not known and should be investigated. Our assumption is that the sediment, because of the weaker adhesion on the plastic conical tubes, easily gets detached from the surface of the conical tube and is removed with the supernatant. Based on our findings we recommend further investigation of the effects of tube material on the quality of urine sediment.

In the analysis of the method of supernatant aspiration, the number of erythrocytes, leukocytes and squamous epithelial cells *per* objective field was found to be significantly lower after the suction of supernatant then after decanting. These results can be explained by the fact that we used non-standardized disposable plastic pipettes and not the recommended adjusted vacuum tool for the suction of the supernatant. Therefore, our routine protocol of decanting supernatant from the urine sediment was kept as the recommended protocol.

Even though urine collection is not an invasive method for patients and volume of urine sample is usually not an issue, sometimes, especially in the paediatric population, very low volume of sample is delivered to laboratory. Therefore, the European Guidelines recommended that urine volumes of 5 mL can also be used for sediment examination. However, our analysis proved that number of erythrocytes, leukocytes, squamous epithelial cells, total non-hyaline casts and the frequency of granular casts were significantly lower in tubes with 5 mL urine sample than in tubes with 10 mL urine sample. These results were expected, but nonetheless concerning. False negative results in smaller volume of urine can lead to serious mistakes in diagnostics, monitoring, management and therapy of renal patients, where getting adequate urine volume is notably difficult (*19*, *20*). Of special concern is the lower number of casts found in 5 mL urine samples, which are specific markers used in diagnosis and monitoring of renal patients (*20*-*23*). According to these results, our laboratory will introduce a change into previously established routine procedure. Since 5 mL of urine samples is clearly not sufficient for an adequate urine sediment analysis, such samples are not acceptable in routine laboratory work.

Recommendations for standardized preparation of urine sediment were published in 2000 (*1*). It is well known that almost two decades is a long time in the fast developing world of technology and diagnostics. Nowadays, time consuming manual microscopy is being replaced by fully automated urine analyzers (*19*, *24*). These systems are quickly becoming part of routine work in both large and small laboratories as a fast way to exclude urine samples from healthy individuals, increase productivity and reduce inter-observer variability (*16*, *19*, *24*, *25*). However, manual examination of urine sediment is still part of routine practice in many European countries. Cwiklinska *et al*. presented results of the Polish external quality assessment program for urinalysis. They showed that only 13% of laboratories use automatic systems for sediment examination. Moreover, even though laboratories are asked to use standardized procedure for sediment examination, only 29% of the results could have been considered to be standardized (16% – manual methods, 13% – automated systems) (*26*).

Although several studies mentioned low levels of adherence to standardized methods, none had directly compared European Guidelines and routine procedures. We have investigated the effect of our modifications and proved that we can use them without compromising patient safety. An update of the Guidelines with more focus on new technologies is sorely needed.

One of the limitations of our study is that all of the samples were examined by the same person which is not a realistic representation of routine work. However, that was done to eliminate the bias that can be created by interpretation of urine sediment results by different people (*21*). Another limitation was the non-standardized equipment we used in supernatant removal. Because of the unavailability of an adjusted vacuum tool we used disposable plastic pipettes with predefined volume of 10 mL which could have affected our results. Moreover, we were limited with the number of available conical tubes, so our sample size in that comparison is noticeably smaller than in the other two comparisons.

In conclusion, all investigated modifications differ significantly from the protocol for urine sediment preparation recommended in European Urinalysis Guidelines. However, careful analysis reveals that if recommendations described in the Guidelines are used (centrifugation at 400xg, suction of supernatant, conical tube or 5 mL of sample), significantly lower number of different sediment elements are observed. In order to avoid false negative findings of the urine sediment investigation, our recommended protocol will include centrifugation at 1358xg, round bottom tube, decanting of supernatant and 10 mL as minimal volume of sample.
